# Diagnostic value of parameters derived from planar MUGA for detecting HFpEF in coronary artery disease patients

**DOI:** 10.1186/s12872-023-03061-w

**Published:** 2023-01-19

**Authors:** Qiaozhi Liu, Shuaishuai Zhou, Qi Wu, Ronghua Zuo, Shengjue Xiao, Xiaotong Wang, Ailin Liu, Jie Liu, Hong Zhu, Defeng Pan

**Affiliations:** 1 Department of Cardiology, Zaozhuang Municipal Hospital, Zaozhuang, 277100 Shandong China; 2 Department of Cardiology, The Affiliated Hospital of Xuzhou Medical University, 99 Huaihai West Road, Xuzhou, 221004 Jiangsu China; 3 Department of Cardiology, The Affiliated Suqian First People’s Hospital of Nanjing Medical University, Xuzhou, 223812 Jiangsu China; 4 Department of Anesthesiology, The First Affiliated Hospital of Nanjing Medical University, Nanjing, 210029 Jiangsu China; 5 Department of Cardiology, School of Medicine, Zhongda Hospital, Southeast University, Nanjing, 210009 Jiangsu China

**Keywords:** Planar gated blood-pool imaging, Heart failure with preserved ejection fraction, Echocardiography, Coronary artery disease, Left ventricular end-diastolic pressure

## Abstract

**Background:**

In recent years, heart failure with preserved ejection fraction (HFpEF) has received increasing clinical attention. To investigate the diagnostic value of diastolic function parameters derived from planar gated blood-pool imaging (MUGA) for detecting HFpEF in coronary atherosclerotic heart disease (coronary artery disease, CAD) patients.

**Methods:**

Ninety-seven CAD patients with left ventricular ejection fraction ≥ 50% were included in the study. Based on the left ventricular end-diastolic pressure (LVEDP), the patients were divided into the HFpEF group (LVEDP ≥ 16 mmHg, 47 cases) and the normal LV diastolic function group (LVEDP < 16 mmHg, 50 cases). Diastolic function parameters obtained by planar MUGA include peak filling rate (PFR), filling fraction during the first third of diastole (1/3FF), filling rate during the first third of diastole (1/3FR), mean filling rate during diastole (MFR), and peak filling time (TPF). Echocardiographic parameters include left atrial volume index (LAVI), peak tricuspid regurgitation velocity (peak TR velocity), transmitral diastolic early peak inflow velocity (E), average early diastolic velocities of mitral annulars (average e′), average E/e′ ratio. The diastolic function parameters obtained by planar MUGA were compared with those obtained by echocardiography to explore the clinical value of planar MUGA for detecting HFpEF.

**Results:**

The Receiver-operating characteristic curve analysis of diastolic function parameters obtained from planar MUGA and echocardiography to detect HFpEF showed that: among the parameters examined by planar MUGA, the area under the curve (AUC) of PFR, 1/3FF, 1/3FR, MFR and TPF were 0.827, 0.662, 0.653, 0.663 and 0.809, respectively. Among the echocardiographic parameters, the AUCs for average e′, average E/e′ ratio, peak TR velocity, and LAVI values were 0.747, 0.706, 0.735, and 0.633. The combination of PFR and TPF showed an AUC of 0.856. PFR combined with TPF value demonstrated better predictive value than average e′ (*Z* = 2.020, *P* = 0.043).

**Conclusion:**

Diastolic function parameters obtained by planar MUGA can be used to diagnose HFpEF in CAD patients. PFR combined with TPF was superior to the parameters obtained by echocardiography and showed good sensitivity and predictive power for detecting HFpEF.

## Background

HFpEF is a specific type of heart failure characterized by diastolic dysfunction, which actually accounts for more than 50% of heart failure patients. CAD accounts for approximately 49.6% of the significant comorbidities of heart failure [1]. The vast majority of patients with CAD often have LV diastolic dysfunction before the development of LV systolic dysfunction [2]. Early detection and accurate assessment of LV diastolic dysfunction are important for patients’ early diagnosis and treatment.

Current guidelines recommend echocardiography for the diagnostic process of HFpEF, with common indices including annular e′ velocity, average E/e′ ratio, LAVI, and peak TR velocity [3, 4]. However, its indexes to assess LV diastolic function should be interpreted in a more informative context, and there is still no single echocardiographic parameter to measure LV diastolic function directly [5]. At the same time, it is highly influenced by the experience and level of the operator. Therefore, it is essential to find a more sensitive and accurate examination method for the early diagnosis of HFpEF.

MUGA has been widely used to assess LV systolic function using several commercial software programs [6]. However, few studies on planar MUGA evaluate LV diastolic function, especially in patients with normal LV systolic function. Therefore, the LVEDP was used as the gold standard for assessing LV diastolic function in this study. Applied diastolic function parameters obtained by planar MUGA in comparison with those obtained by echocardiography to evaluate the sensitivity, specificity and accuracy of planar MUGA parameters for detecting HFpEF, and to explore the clinical value of planar MUGA, which is beneficial to the early diagnosis and treatment of LV diastolic dysfunction in patients with CAD, thus benefiting patients.

## Methods

### Subjects

A retrospective cohort analysis was used for this study. Ninety-seven patients diagnosed with CAD at the Department of Cardiovascular Medicine in the Affiliated Hospital of Xuzhou Medical University from September 2021 and January 2022 were selected. LVEDP ≥ 16 mmHg (1 mmHg = 0.133 kPa) was defined as increased LV filling pressure [7, 8]. Based on LVEDP, patients were divided into the HFpEF group (LVEDP ≥ 16 mmHg, 47 cases) and the normal LV diastolic function group (LVEDP < 16 mmHg, 50 cases). The Medical Ethics Committee approved the study of the Affiliated Hospital of Xuzhou Medical University (Number: XYFY2021-KL164-01).

Inclusion criteria: (1) All patients with suspected or known coronary artery disease who have completed echocardiography, planar MUGA and cardiac catheterization 1–3 days after admission and whose diagnosis of CAD was confirmed by coronary arteriography with a subepicardial coronary artery diameter stenosis more than 50% [9]; (2) sinus rhythm; (3) hemodynamic stability; (4) LVEF ≥ 50% (based on left ventriculography findings) .

Exclusion criteria: pulmonary heart disease (10), congenital heart disease (1), rheumatic heart disease (0), cardiomyopathy and pericardial disease (2), hyperthyroidism (2), arrhythmias (5), severe anemia (0), severe hepatic and renal dysfunction (1), LVESV′ < 20 mL measured by planar MUGA (3) (because assessment of LV volume and function is less accurate in very small volume patients [6, 10]).

## Echocardiography

**Fig. 1:**
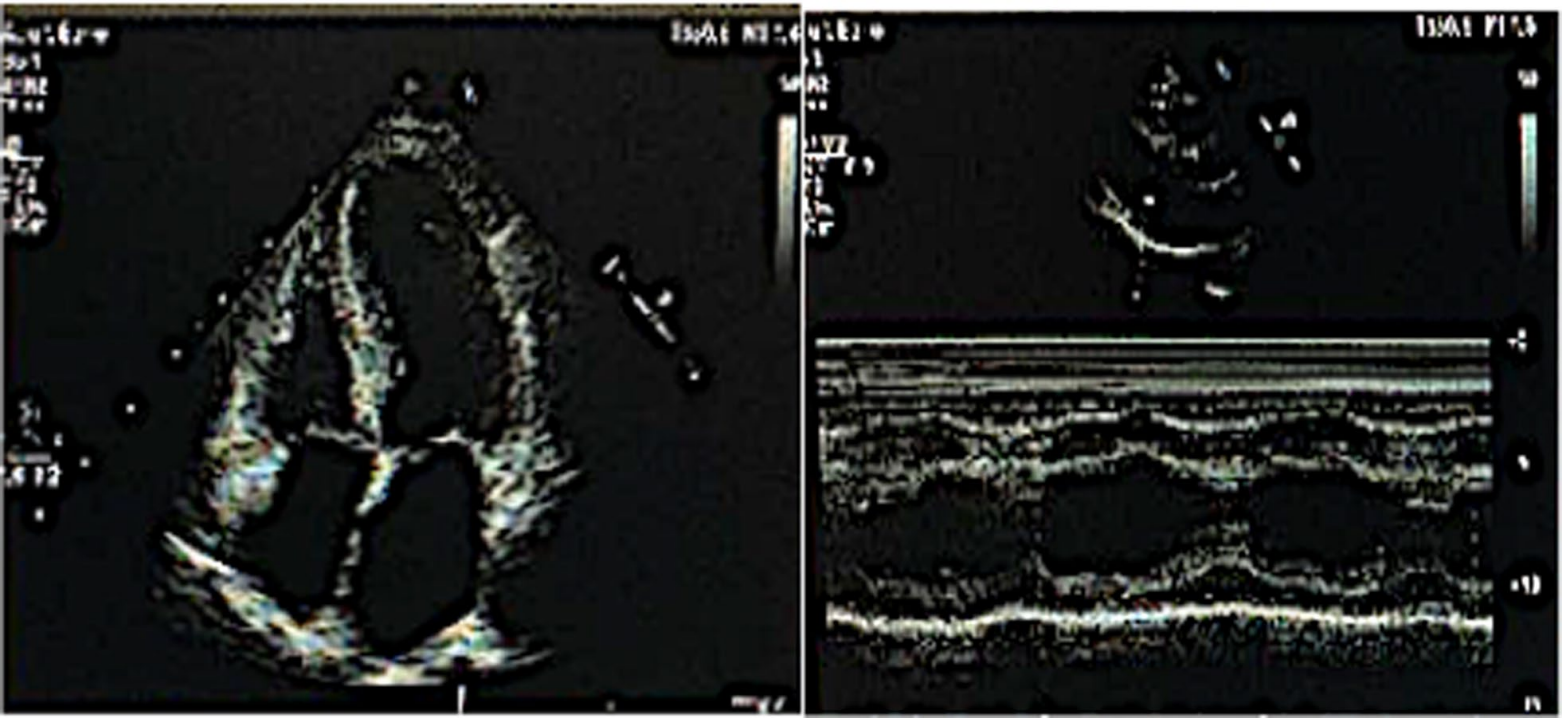
Image acquisition for colour Doppler echocardiography

The Philips EPIQ 7 C colour Doppler echocardiography was used for image acquisition. It acquired 4-chamber, 2-chamber, and 3-chamber cardiac dynamic images of the apical region to measure left atrial volume index (LAVI), LV ejection fraction(EF), and peak tricuspid regurgitation(TR)velocity. The sample volume was placed at the level of the mitral valve apex in the apical 4-chamber view to measure transmitral diastolic early peak inflow velocity (E). A pulsed tissue Doppler imaging procedure was initiated in the 4-chamber apical view to place the sample volume at the level of the mitral valve ring in the left ventricular lateral wall and the ventricular septum, respectively, to measure early mitral annular diastolic motion velocity (e′). The average e′ and average E/e′ ratio were calculated. Partial image analysis of echocardiography is shown in Fig. 1.

### Planar MUGA

**Fig. 2:**
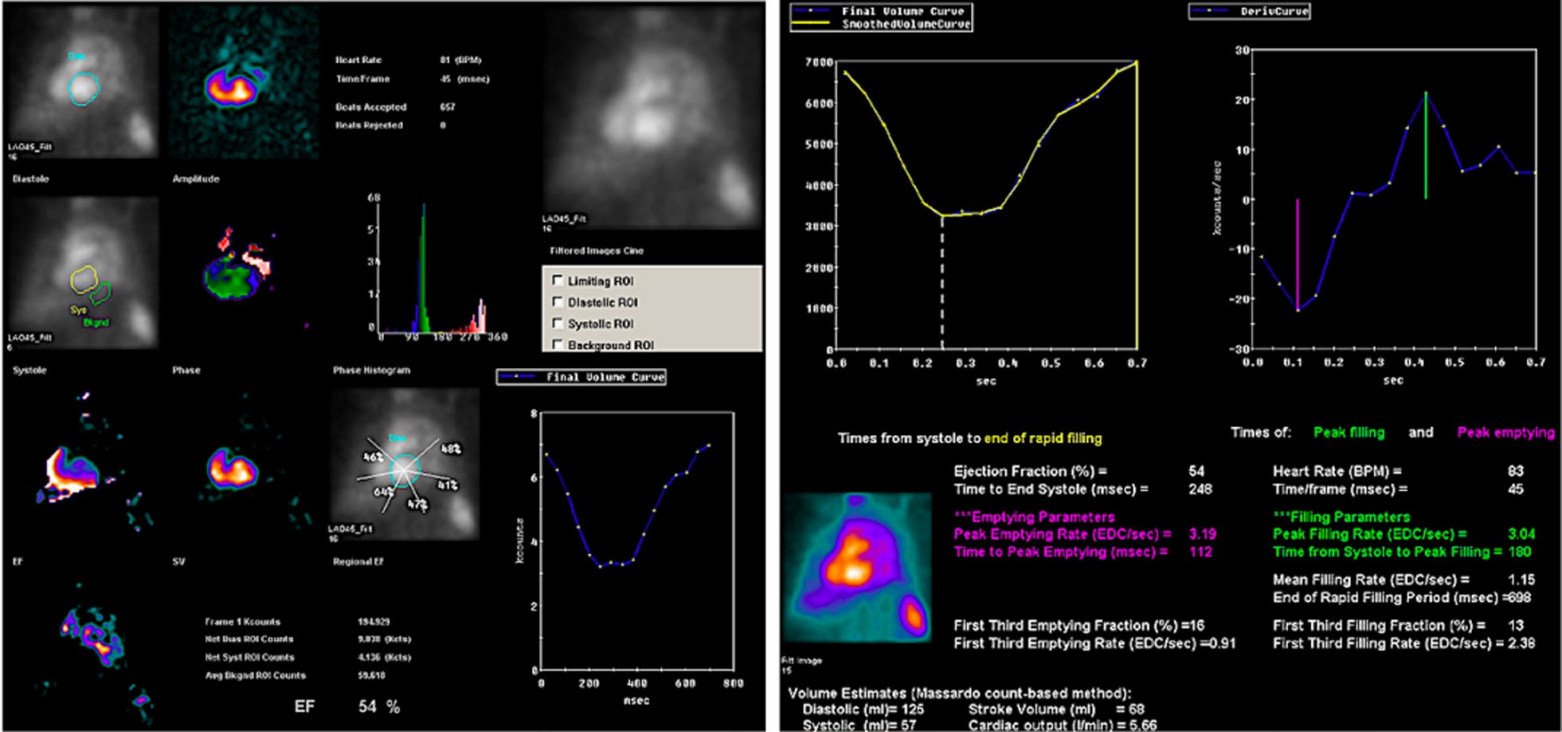
Planer MUGA image analysis and parameter report

The imaging equipment was a GE Infinia VC Hawkeye II variable-angle dual-detector SPECT/CT with a parallel-hole low-energy high-resolution collimator, 140 KeV energy peak, 20% window width selection, 64 × 64 acquisition matrix, and Zoom 3.0. Using the in vivo nucleotide labeling method, patients were first given 1–2 intravenous injections of stannous pyrophosphate (PYP, Jiangsu Atomic Medical Research Institute Jiang Yuan Pharmaceutical Factory) (based on 20ug/kg). And 20 min later, 99mTcO4 eluate 740-925MBq (20-25mCi, Atomic Hi-Tech Co.) was injected intravenously, and the examination started after 20–30 min. The patient was placed in the supine position, and the patient was connected to the ECG level, connected to the gating device, and observed to show a good signal. The detector was routinely acquired in anterior position, 30°–45° left anterior oblique position (to distinguish the left and right blood pools), 70° left anterior diagonal position, and other parts were added if necessary. Sixteen frames per cardiac cycle were preset for acquisition, 400–600 cardiac cycles were preset for investment, and the total preset acquisition count was 3000k counts. The Xeleris program was utilized for quantitative analysis of the images to obtain cardiac function parameters: LV ejection fraction (LVEF’), peak filling rate (PFR), filling fraction during the first third of diastole (1/3FF), filling rate during the first third of diastole (1/3FR), mean filling rate during diastole (MFR), and peak filling time (TPF). Planer MUGA image analysis and parameter report are shown in Fig. 2.

### Coronary arteriography examination and cardiac catheterization

**Fig. 3:**
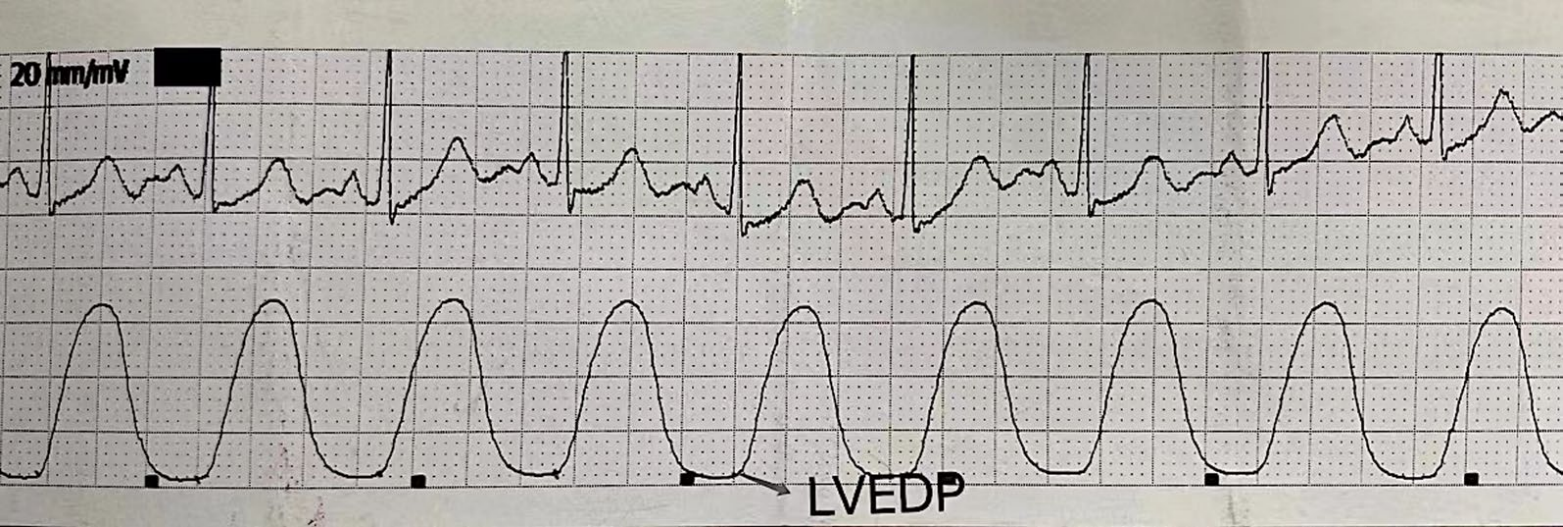
Left ventricular pressure curve

Left coronary artery and right coronary artery angiography were performed sequentially using the Judkins method. Then the 6 F pigtail catheter was placed into the left ventricle, the pressure transducer was connected, and a Mindray monitor was applied to record the II-lead ECG, the left ventricular pressure curve (as shown in Fig. 3) simultaneously, and the recording speed was 25 mm/s. The left ventricular pressure point corresponding to the onset of the QRS wave group of the ECG was the LVEDP (taken from the mean value of LVEDP measured during five consecutive cardiac cycles). Left ventriculography was performed after completion of the above operations. A 6 F pigtail catheter was used to inject 30–40 ml of contrast medium at a 15 ml/s using a high-pressure syringe. The same sinus cardiac cycle was selected without premature beats, LVEDV and LVESV were measured, and the calculation of LVEF= (LVEDV-LVESV)/LVEDV, the volume was calculated according to the two-plane Simpson method.

### Statistical analysis

SPSS 25.0 and MedCalc 20.106 software were used to process the data. The measurement data of normal distribution were expressed as mean ± standard Deviation (mean ± SD), and the measurement data of nonnormal distribution were described as M (Q1, Q3). The independent sample t-test was used to compare the measurement data of normal distribution between the 2 groups. Non-normal distribution data using nonparametric test (the Mann–Whitney U test). Pearson correlation analysis was used for correlation analysis of two variables conforming to a normal distribution; Spearman correlation analysis was used for nonnormal distribution. Applied ROC curves to evaluate the predictive value of each parameter of planar MUGA and echocardiography for detecting HFpEF. The area under the curve was compared using the Delong test. *P* < 0.05 was considered statistically significant.

## Results

### Comparison of general data between the two groups

In this study, 97 patients met the inclusion criteria, including 47 cases in the HFpEF group and 50 cases in the normal LV diastolic function group. There were statistically significant differences in gender, diabetes, hypertension, multivessel lesions and age between the two groups (*P* < 0.05), while there were no statistically significant differences in other data (*P* > 0.05), as shown in Table 1.

**Table 1:** Comparison of general information between the two groups of patients

Projects	The HFpEF group (n = 47)	The normal LV diastolic function group (n = 50)	t/z/χ2	*P*-value
Male/case (%)	22 (46.8)	36 (72.0)	6.395	0.011
Diabetes/case (%)	32 (22.8)	15 (24.2)	14.07	< 0.001
Hypertension/case (%)	29 (61.7)	19 (24.7)	5.445	0.02
Multivessel lesions/case (%)	39 (83.0)	31 (62.0)	5.308	0.021
Smoking/case (%)	11 (23.4)	15(30.0)	0.537	0.464
Alcohol consumption/case (%)	9(19.1)	8(16.0)	0.166	0.684
Age/Years	66.60 ± 8.21	59.36 ± 10.45	3.775	< 0.001
SBP (mmHg)	137.36 ± 16.69	133.88 ± 16.63	1.029	0.306
DBP (mmHg)	77.06 ± 9.70	80.06 ± 8.88	− 1.588	0.116
HR (times/min)	68.55 ± 8.42	69.78 ± 9.19	− 0.684	0.496
BMI (kg/m^2^ )	25.55 ± 3.61	25.17 ± 3.65	0.507	0.614
GFR (ml/min)	103.01 ± 13.33	97.88 ± 18.94	1.067	0.291
Fasting blood glucose (mmol/l)	5.85 ± 1.41	6.24 ± 1.79	− 1.111	0.270
Hemoglobin (g/l)	130.00 ± 14.43	134.88 ± 15.15	− 1.613	0.11
White blood cell count (10^9/l)	6.10 ± 1.89	6.38 ± 1.69	− 0.773	0.442
CHOL (mmol/l)	3.86 ± 1.09	3.79 ± 0.92	0.346	0.730
HDL-C (mmol/l)	0.97 ± 0.24	0.97 ± 0.29	0.102	0.919
LDL-C (mmol/l)	2.14 ± 0.82	2.23 ± 0.78	− 0.562	0.576
TG (mmol/l)	1.49 (0.99, 2.45)	1.26 (0.87, 2.35)	− 0.586	0.558
Lipoprotein a (mg/l)	237.00 (180.50, 343.00)	282.00 (154.00, 375.00)	− 0.477	0.634
C-reactive protein (mg/l)	1.40 (0.70, 4.55)	1.40 (0.50, 4.50)	− 0.586	0.558
NT-ProBNP (Pg/ml)	103.00 (74.35, 402.00)	87.20 (48.60, 173.00)	− 1.946	0.052
hsTnT (ng/l)	8.84 (6.81, 16.54)	7.80 (6.13, 15.00)	− 0.092	0.927

*SBP* Systolic blood pressure, *DBP* Diastolic blood pressure, *HR* Heart rate, *BMI* Body mass index, *GFR* Glomerular filtration rate, *CHOL* Total cholesterol, *HDL-C* High-density lipoprotein cholesterol, *LDL-C* Low-density lipoprotein cholesterol, *TG* Triglycerides; NT-ProBNP, Brain natriuretic peptide precursor (N-terminal), hsTnT, ultrasensitive troponin T

## Comparison of diastolic function parameters function between the two groups

TPF, peak TR velocity, average E/e′ ratio and LAVI were significantly higher in the HFpEF group patients than in the normal LV diastolic function group (*P* < 0.05), LVEF’, PFR, 1/3FR, 1/3FF, MFR, average e′ were significantly lower than those of the normal LV diastolic function group (*P* < 0.05), and the differences in LVEDV′, LVESV′, EF, LVEDV, LVESV and LVEF were not statistically significant between the two groups (*P* > 0.05), as shown in Table 2.

**Table 2:** Comparison of cardiac function parameters between two groups of patients

Parameters	The HFpEF group (n = 47)	The normal LV diastolic function group (n = 50)	t/z	*P*-value
*Planar MUGA*				
LVEF′ (%)	56.96 ± 7.46	60.36 ± 8.37	− 2.111	0.037
1/3FR (EDC/s)	1.75 ± 0.61	2.03 ± 0.52	− 2.456	0.016
TPF (ms)	177.96 ± 35.87	149.90 ± 29.63	4.211	< 0.001
PFR (EDC/s)	2.11 (1.81, 2.66)	2.65 (2.28, 2.86)	− 3.657	< 0.001
1/3FF (%)	33.00(25.00, 38.00)	39.00 (30.50, 45.25)	− 2.748	0.006
MFR (EDC/s)	1.25 (1.03, 1.38)	1.36 (1.23, 1.54)	− 2.769	0.006
LVEDV′ (ml)	101.00 (84.00, 117.00)	101.00 (87.00, 125.00)	− 0.567	0.571
LVESV′ (ml)	41.00 (32.00, 54.00)	38.00(34.00, 51.00)	− 0.419	0.675
*Echocardiography*				
Peak TR velocity (m/s)	2.58 ± 0.22	2.41 ± 0.15	4.508	< 0.001
Average e′ (m/s)	0.07 (0.06, 0.08)	0.09 (0.08, 0.10)	− 4.968	< 0.001
Average E/e′ ratio	13.98 (12.29, 14.89)	12.84 (10.53, 13.89)	− 3.494	< 0.001
LAVI (ml/m^2^)	24.79 (20.98, 31.57)	22.67 (20.27, 26.56)	− 2.252	0.024
EF (%)	60.00 (59.00, 64.00)	63.00 (59.00, 66.25)	− 1.720	0.085
*Cardiac catheterization*				
LVEF (%)	63.51 ± 7.80	65.74 ± 10.14	− 1.211	0.229
LVEDV (ml)	100.00 (87.00, 135.80)	104.50 (85.00, 154.75)	− 0.834	0.404
LVESV (ml)	36.00 (29.20, 52.00)	36.00 (29.00, 47.00)	− 0.148	0.882

## ROC curve analysis

**Fig. 4:**
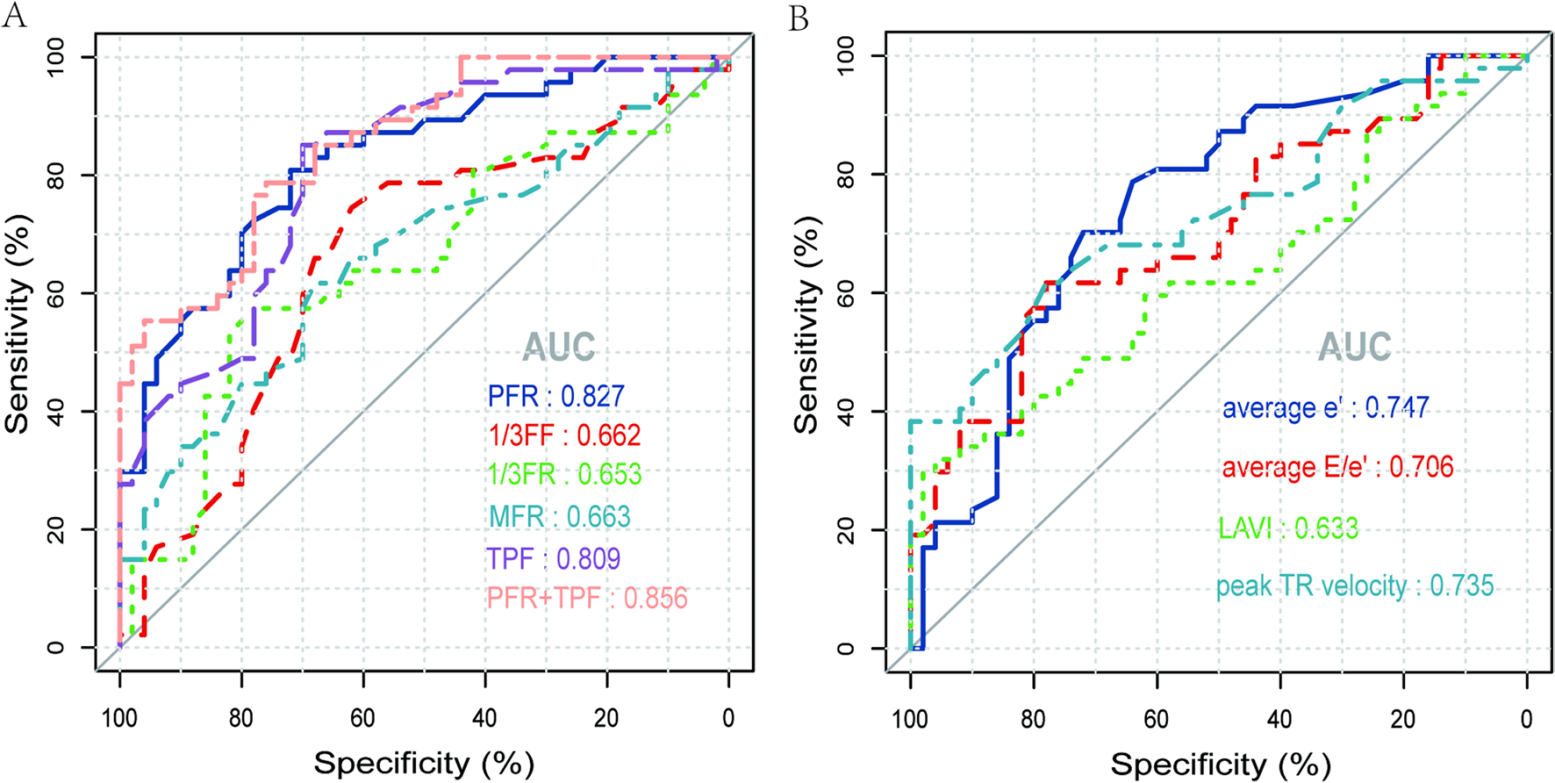
ROC curves of planar MUGA (Figure A) and echocardiography-derived (Figure B) parameters for detecting HFpEF

Among the parameters examined by planar MUGA, the ROC curves for detecting HFpEF with PFR, 1/3FF, 1/3FR, MFR and TPF are shown in Table 3; Fig. 4 A. The areas under the curve (AUCs) for these values were 0.827, 0.662, 0.653, 0.663 and 0.809, respectively. These values showed sensitivities of 81%, 75%, 57%, 62%, and 85%, respectively, and specificities of 72%, 62%, 78%, 68%, and 70%, respectively, for correctly identifying patients with HFpEF.

Among the echocardiographic parameters, the ROC curves for detecting HFpEF with average e′, average E/e′ ratio, peak TR velocity, and LAVI are shown in Table 3; Fig. 4B. The AUCs for these values were 0.747, 0.706, 0.735, and 0.633. The sensitivity of these values was 78%, 62%, 62%, and 32%, respectively, and the specificity was 64%, 78%, 78%, and 96%, respectively, for correctly identifying patients with HFpEF .

The area under the ROC curve for average e′ detecting HFpEF was the highest among echocardiographic parameters. However, the AUC for both PFR and TPF was higher than the average e′ among the parameters obtained by planar MUGA. Delong’s test found no statistically significant difference between the AUCs of PFR and average e′ and TPF and average e′ (*P* > 0.05). In the present study, the combination of PFR and TPF revealed an AUC of 0.856, higher than the predictive value of both indices alone. The Delong test yielded a statistically significant difference between the combination of PFR and TPF compared with the AUC of average e′ (*Z* = 2.020, *P* = 0.043), indicating that the accuracy of PFR combined with TPF in detecting HFpEF is better than that of individual echocardiographic parameters.

**Table 3:** ROC curve of planar MUGA and echocardiographic-derived parameters for detecting HFpEF

Parameters	Cutoff value(s)	Sensitivity (%)	Specificity (%)	Youden index	95% CI	*P*	AUC
*Planar MUGA*							
PFR (EDC/s)	2.315	81	72	0.529	0.747–0.908	< 0.001	0.827
1/3FF (%)	37.500	75	62	0.365	0.551–0.772	0.006	0.662
1/3FR (EDC/s)	1.750	57	78	0.354	0.542–0.764	0.009	0.653
MFR (EDC/s)	1.275	62	68	0.297	0.554–0.772	0.006	0.663
TPF (ms)	152.500	85	70	0.551	0.723–0.894	< 0.001	0.809
PFR + TPF		79	76	0.547	0.785–0.927	< 0.001	0.856
*Echocardiography*							
Average e′ (m/s)	0.0815	78	64	0.427	0.649–0.846	< 0.001	0.747
Average E/e′ ratio	13.895	62	78	0.397	0.602–0.809	< 0.001	0.706
Peak TR velocity (m/s)	2.545	62	78	0.397	0.634–0.835	< 0.001	0.735
LAVI (ml/m^2^)	29.575	32	96	0.279	0.521–0.745	0.024	0.633

## Correlation analysis

The correlation between diastolic function parameters obtained by planar MUGA and echocardiography, and LVEDP is shown in Table 4.

Among the parameters examined by planar MUGA, 1/3FF, 1/3FR, and MFR had low correlation with LVEDP (*r* = − 0.212, − 0.354 and − 0.305, *P* < 0.05). However, there were significant correlations between PFR, TPF, PFR combined with TPF and LVEDP (*r* = − 0.589, 0.593, 0.650, *P* < 0.001).

Among the echocardiographic parameters, correlations between average e′, peak TR velocity, LAVI and LVEDP were lower (*r* = -0.377, 0.246, 0.256, *P* < 0.05) and correlations between average E/e′ ratio and LVEDP were improved (*r* = 0.544, *P* < 0.001).

In this study, the correlation between the average E/e′ ratio and LVEDP was the best among the diastolic function parameters obtained by echocardiography. We did a correlation analysis between diastolic functional parameters obtained by planar MUGA and the average E/e’ ratio, showing that there was some correlation between PFR, MFR, TPF, PFR combined with TPF and the average E/e′ ratio, but the correlation was not highly (*r* = − 0.334, − 0.266, 0.310, 0.401, *P* < 0.05), as shown in Table 5.

**Table 4:** Correlation analysis between planar MUGA, echocardiographic-derived parameters and LVEDP

Parameters	LVEDP	
	*r*	*P*
*Planar MUGA*		
PFR (EDC/s)	− 0.589	< 0.001
1/3FF (%)	− 0.212	0.037
1/3FR (EDC/s)	− 0.354	< 0.001
MFR (EDC/s)	− 0.305	0.002
TPF (ms)	0.593	< 0.001
PFR + TPF	0.650	< 0.001
*Echocardiography*		
Average e′ (m/s)	− 0.377	< 0.001
Average E/e′ ratio	0.544	< 0.001
Peak TR velocity (m/s)	0.246	0.015
LAVI (ml/m^2^)	0.256	0.011

**Table 5:** Correlation analysis between average E/e’ ratio and each parameter

Parameters	Everage E/e’ ratio	
	*r*	*P*
Age/years	0.178	0.080
Body mass index (kg/m^2^)	− 0.090	0.388
Systolic blood pressure (mmHg)	0.072	0.486
Diastolic blood pressure (mmHg)	0.090	0.383
Heart rate (times/min)	0.050	0.963
PFR (EDC/s)	− 0.334	0.001
1/3FF (%)	− 0.065	0.527
1/3FR (EDC/s)	− 0.095	0.354
MFR (EDC/s)	− 0.266	0.009
TPF (ms)	0.310	0.002
PFR + TPF	0.401	< 0.001

## Discussion

CAD is one of the most common cardiovascular diseases, and its end-stage outcome is often heart failure. It has been shown that patients with CAD have a significantly higher incidence of LV diastolic dysfunction and significantly lower long-term survival than patients with normal LV diastolic function [1, 11]. The prevalence of HFpEF is increasing yearly, the pathophysiological mechanism is complex, the diagnostic process is cumbersome, and the clinical diagnosis and treatment are extremely challenging. Therefore, early assessment of LV diastolic function in patients has important guiding values for clinical treatment and prognosis of patients.

Current guidelines recommend that the main parameters commonly used to assess LV diastolic function by echocardiography are e′, E/e′, LAVI, and peak TR velocity [5]. e′ is primarily determined by LV relaxation [12], and the average e′ is the average early diastolic velocities of mitral annulars. Because it is measured at multi-sectional and multiple points, a change in any of these points will change the average e′, so it is thought that it can be a more sensitive assessment of left ventricular diastolic function. The E/e′ ratio is associated with LV chamber stiffness and fibrosis and can be used to assess LV filling pressures [12]. LAVI > 34 ml/m^2^ is a key structural change in ejection fraction preserved heart failure and an independent predictor of atrial fibrillation, heart failure, and even death, and can be used as an index for clinical evaluation of LV diastolic function [13, 14]. Peak TR velocity reflects pulmonary artery pressure and is an indirect marker of LV diastolic dysfunction [5].

NT-proBNP is the preferred serum marker recommended by heart failure guidelines and is widely used for preventive screening, efficacy testing, prognosis assessment, and risk stratification in heart failure patients. However, diastolic heart failure may also exist with normal NT-proBNP. A recent study published in the European Heart Journal showed that 60% of patients with HFpEF had normal NT-proBNP levels (< 125 ng/L)[15]. Such patients had an increased rate of mortality or rehospitalization for heart failure compared with those without heart failure. In this study, although NT-proBNP levels were not significantly different between patients with HFpEF and those with normal diastolic function, the patients still had diastolic heart failure as indicated by a combination of several tests. Larger study sample sizes are needed to further verify this in the future.

In a study related to the assessment of LV diastolic function by quantitative gated myocardial perfusion SPECT, Nakae et al. [16] measured 1/3FF, PFR, and TPF by SPECT and showed that TPF was elevated and 1/3FF and PFR were decreased in elderly people older than 60 years of age, allowing the evaluation of left ventricular diastolic dysfunction. The results of this study showed that the values of 1/3FR, PFR, 1/3FF and MFR were lower in patients with HFpEF than in patients with normal LV diastolic function, whereas the values of TPF were higher than in patients with normal LV diastolic function, similar to the results of Nakae et al.

Patel et al. [17] showed that the area under the ROC curve for parameters PFR, TPF, and 1/3FR measured by Myocardial Perfusion Imaging was 0.83, 0.75, and 0.80 for detecting LVEDP ≥ 18 mmHg, respectively, and that PFR, TPF, and 1/3FR were moderately correlated with LVEDP (*r* = − 0.53, 0.45, − 0.45, *P* < 0.01). Zhang Fen et al. [18]showed that good accuracy of LAVI, peak TR velocity, lateral e′, average e′, septal E/e′, and average E/e′ in diagnosing LVEDP elevation (AUC between 0.7 and 0.9). Previous studies have applied two examination methods to compare and evaluate LV systolic or diastolic function. In comparison, the present study used three methods to compare and assess LV diastolic function, namely planar MUGA, echocardiography and the gold standard cardiac catheterization method, to investigate the clinical application value of planar MUGA to evaluate LV diastolic function.

First, we analyzed the ROC curves of diastolic function parameters obtained by planar MUGA and echocardiography to predict LVEDP ≥ 16mmHg, suggesting that the AUCs of PFR and TPF were higher among the planar MUGA parameters 0.827 and 0.809, respectively. The sensitivities of these two parameters were 81% and 85%, respectively, and the specificities were 72% and 70% for predicting LVEDP ≥ 16mmHg, respectively. Among the echocardiographic parameters, the average e’ had a relatively high AUC of 0.747, with a sensitivity of 78% and a specificity of 64% for predicting LVEDP ≥ 16mmHg, respectively. The accuracy of PFR combined with TPF in predicting LVEDP ≥ 16mmHg is better than that of echocardiography alone. Secondly, we applied a correlation analysis between diastolic function parameters obtained by planar MUGA and echocardiography and LVEDP, suggesting a better correlation between PFR, TPF, PFR combined with TPF, and LVEDP, which is in line with Patel et al.Among the echocardiographic parameters, the correlation between the average E/e’ ratio and LVEDP was better, which is similar to the results of previous studies [19]. These parameters may provide a good screen individually and, in combination, would be a powerful tool to assess LV diastolic function. Although echocardiography is more available and does not use radiation, our study demonstrates that PFR combined with TPF is superior to the parameters obtained by echocardiography and shows good sensitivity and predictive power for detecting HFpEF. In the future, we will conduct larger trials to determine the effectiveness of planar MUGA and to better guide its clinical application.

Yamano et al. [20] compared LV diastolic function parameters measured by gated single-photon emission tomography with colour Doppler ultrasound, suggesting a moderate correlation between PFR and E, a significant correlation with E/e’ ratio. Malek et al. [21] showed that myocardial perfusion imaging can be used as a highly specific means of detecting LV diastolic dysfunction compared with echocardiography. In the present study, we applied a correlation analysis between diastolic functional parameters obtained by planar MUGA and the average E/e’ ratio, showing that there was some correlation between PFR, TPF, PFR combined with TPF, and the average E/e’ ratio, but the correlation coefficients obtained in this study were small and larger sample size is needed of studies for further validation.

## Limitations

All the subjects of this study were patients with LVEF ≥ 50%, LV diastolic function in patients with LVEF < 50% will be further explored in the future. The optimal thresholds derived in the study were only applicable to the category presented in the manuscript and not to other populations. This study used only LVEDP ≥ 16 mmHg at rest as the gold standard for assessing HFpEF, and will continue to be studied by considering the inclusion of exercise metrics in the future. This study did not explore echocardiographic combinations and therefore cannot exclude that echocardiography may have similar or superior accuracy in a comparable analysis.

## Conclusion

Diastolic function parameters obtained by planar MUGA can be used to diagnose HFpEF in CAD patients. PFR combined with TPF was superior to the parameters obtained by echocardiography and showed good sensitivity and predictive power for detecting HFpEF.

## Data Availability

Data are available on request due to privacy or other restrictions. If someone wants to request the data from this study, they can contact Qiaozhi Liu.
